# Microglia Purinoceptor P2Y6: An Emerging Therapeutic Target in CNS Diseases

**DOI:** 10.3390/cells9071595

**Published:** 2020-07-01

**Authors:** Shehata Anwar, Vincent Pons, Serge Rivest

**Affiliations:** 1 Neuroscience Laboratory, CHU de Québec Research Center (CHUL), Department of Molecular Medicine, Faculty of Medicine, Laval University, Québec City, QC G1V 0A6, Canada; shehata-ibrahim-anwar.shehata.1@ulaval.ca (S.A.); vincent.pons.1@ulaval.ca (V.P.); 2 Department of Pathology, Faculty of Veterinary Medicine, Beni-Suef University, Beni-Suef 62511, Egypt

**Keywords:** P2Y6R, microglia, phagocytosis, neuroinflammation, pro-inflammatory cytokines

## Abstract

The purinergic receptor P2Y6 is expressed in immune cells, including the microglia that are implicated in neurological disorders. Its ligand, UDP, is a signaling molecule that can serve as an “find-me” signal when released in significant quantities by damaged/dying cells. The binding of UDP by P2Y6R leads to the activation of different biochemical pathways, depending on the disease context and the pathological environment. Generally, P2Y6R stimulates phagocytosis. However, whether or not phagocytosis coincides with cell activation or the secretion of pro-inflammatory cytokines needs further investigation. The current review aims to discuss the various functions of P2Y6R in some CNS disorders. We present evidence that P2Y6R may have a detrimental or beneficial role in the nervous system, in the context of neurological pathologies, such as ischemic stroke, Alzheimer’s disease, Parkinson’s disease, radiation-induced brain injury, and neuropathic pain.

## 1. Introduction

**Figure 1:**
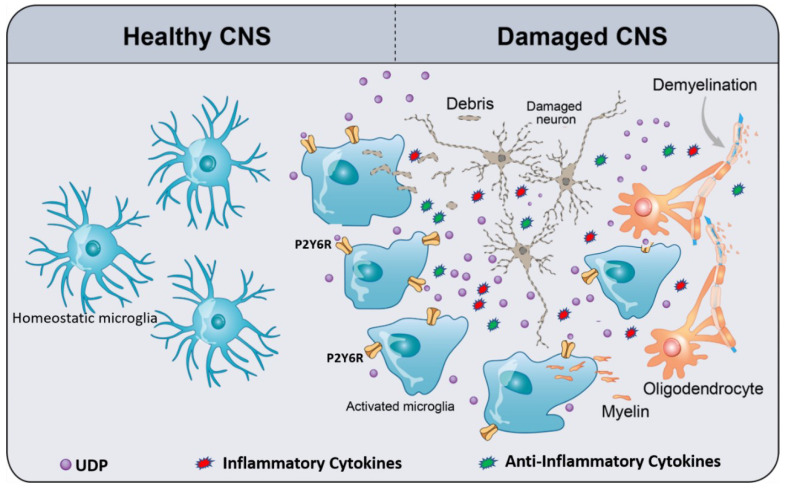
Schematic of UDP/P2Y6R signaling, mediating microglial phagocytosis and chemotaxis. In the healthy CNS, homeostatic microglia (resting microglia) constantly survey and scan the cerebral microenvironment by continuously extending and retracting their ramified processes for the early recognition of damage-associated molecular patterns (DAMPs) such as ATP. Damaged CNS elements (neurons and oligodendrocytes) release or leak ATP/UTP. Furthermore, UTP is easily converted to UDP by ectonucleotidases. UDP acts as an “find-me” signal for P2Y6R. UDP/P2Y6R activates microglia, activated microglia move to the damaged area, and then subsequently recognize UDP as “find-us” signal, attached to the targets, and engulf them. As a result of microglia activation, they release a plethora of inflammatory and anti-inflammatory cytokines depending on the context of the disease. The types and levels of released cytokines differ from a disease to another. The context of a specific disease determines the harmful or the beneficial effects of microglia activation.

Microglia are the myeloid cells of the central nervous system (CNS) [1]. Although their source has been a discussion point for several years, microglia are currently referred to as CNS tissue-resident macrophages, which originate exclusively from the embryonic yolk sac [2,3]. Microglia are long-lived and self-maintaining cells that can survive for years, if not the entire lifespan of an individual, without contribution from the fetal liver or definitive hematopoiesis [4]. Under pathological conditions, microglia substantially change their phenotypes and produce several chemoattractant substances favoring the recruitment of monocytes/macrophages from the circulation [5]. Microglia are distributed throughout the CNS and are in close contact with other CNS cells [6]. For a healthy brain, dynamic crosstalk between microglia and neurons, astroglia, and oligodendrocytes is essential, as microglia not only serve as immune sentinels that protect against infection and inflammation, but also maintain CNS homeostasis from early development through adulthood and senescence [7]. Microglia actively monitor and scan the cerebral microenvironment by continually extending and retracting ramified processes for molecular indicators of damage-associated molecular patterns (DAMPs) and pathogen-associated molecular patterns (PAMPs) [8,9] (Figure 1). Following stimulation in response to injury or pathogen invasion, microglia transform from a surveillant to an activated type involving changes in morphology and gene transcription [10]. Once danger signals are detected, microglia initiate a series of responses triggered by a plethora of surface receptors, such as Toll-like receptors (TLRs), purinergic receptors, scavenger receptors, and cytokine and chemokine receptors [7]. Consequently, the activation of microglia results in the phagocytosis of pathogens, misfolded proteins and dying cells, tissue repair and remodeling, and the recruitment of other peripheral blood immune cells (Figure 1). Recently, it is clear that microglia are implicated in many, if not all, CNS diseases, such as Alzheimer’s disease (AD) [7], multiple sclerosis (MS) [11], Parkinson’s’ disease (PD) [12], Stroke [13], frontotemporal dementia (FTD) [14] and CNS tumors [15], as well as viral, fungal, protozoal, and prionic CNS infections [16,17]. Although microglia contribute to the majority of neurodegenerative diseases, the pathways for their activation and possible contributions to CNS damage continue to be an active subject of research. The activation of purinergic receptors is linked to the movement of microglial processes and chemotaxis in the context of pathology. The purinoreceptors also modulate phagocytosis and the release of cytokines, nitric oxide, and superoxide, which are essential in a pathological response [18].

## 2. Purinergic Signaling

In 1972, Burnstock introduced the “purinergic hypothesis,” which indicated that Adenosine 5′-trisphosphate (ATP) could be released under various conditions and that exogenously delivered ATP could initiate physiological responses [19]. ATP and other purine and pyrimidine nucleotides are released from non-adrenergic, non-cholinergic nerve endings, and non-secretory tissues in response to cell injury [20]. The released nucleotides further control cellular functions by the triggering of membrane-bound purinergic receptors [21]. Purinergic receptors were first identified in 1976 [22]. Two years later, two classes of purinoceptors were identified, defining P1 and P2 adenosine and ATP/ADP, respectively [23]. Based on the molecular structure and transduction mechanisms, there are two subgroups of P2 receptor subgroups—the ionotropic P2X receptors that bind ATP and metabotropic P2Y receptors capable of binding ATP, ADP, UTP, and uridine 5′ diphosphate (UDP) [24]. Ionotropic P2X receptors (P2XRs) are ligand-gated ion channels. Seven P2X subunits (P2X1–P2X7) have been cloned, generating seven different receptors, from which only P2X1R, P2X2R, P2X3R, P2X4R, and P2X7R are functional [25]. Eight mammalian P2Y receptor subtypes (P2Y 1, P2Y 2, P2Y 4, P2Y 6, P2Y 11, P2Y 12, P2Y 13, and P2Y 14) were known as membrane-bound and G-protein-coupled receptors (GPCRs) for extracellular nucleotides [26]. Purinergic signaling plays a leading part in the regulation of various physiological processes, including the recruitment of immune cells, inflammation, and neurotransmission [27,28]. Recently, the dysregulation of purinergic pathways has been correlated with well-defined neurodegenerative and neuroinflammatory disorders [29].

## 3. Microglia P2Y6R

Microglia express multiple functional purinergic receptors, including the P2Y6 receptor (P2Y6R). P2Y6R is widely distributed in various tissues, including the CNS [30,31]. In the CNS, P2Y6R is principally expressed on microglia, non-parenchymal macrophages, and blood-derived monocytes, and P2Y6R chiefly contributes to microglia activation and phagocytosis [31]. P2Y6R has received attention as an essential regulator of inflammation and phagocytosis, and the stimulation of this receptor with its endogenous ligand UDP can trigger the production and the release of a vast plethora of cytokines and chemokines [32]. Herein, we shall present a broad, comprehensive overview of microglial P2Y6R, their pathophysiology in the CNS, and their potential target to treat inflammatory-related neurodegenerative diseases.

## 4. Pathophysiology and Therapeutic Potential of P2Y6R

Little information is available on signals mediated by the P2Y6R [33]. UDP, which acts as an “eat-me” signal for P2Y6R, selectively activates P2Y6R, and later performs its response through the phospholipase C (PLC) signaling pathway. This triggers inositol trisphosphate (IP_3_), which then induces a steady accumulation of intracellular Ca^2+^ levels [34,35] and microglia activation. The endogenous P2Y6R agonist UDP can be leaked from damaged CNS elements to mediate the microglial phagocytosis of myelin debris and apoptotic neurons via P2Y6R (Figure 1) [36,37]. P2Y6R is up-regulated when neurons are damaged, and would function as a sensor for phagocytosis by sensing diffusible UDP signals [37,38]. The inhibition of P2Y6R impairs microglial phagocytosis and worsens radiation-induced brain damages via blocking microglial phagocytosis [39]. Microglia are also unable to clear amyloid deposition after P2Y6R inhibition in 3–4 months old 5xFAD transgenic mouse model Alzheimer’s disease (AD) [40]. In addition, P2Y6R modulates the innate immune reaction and recruitment of monocytes/macrophages in response to invading pathogens into the CNS [41]. These infiltrating monocytes/macrophages are essential for the clearance and phagocytosis of invading bacteria [41]. Microglia promptly acquire properties of reactive subtype, antigen presentation, phagocytosis, and the production of inflammatory mediators, including interleukin 1 (IL-1), IL-6, and tumor necrosis factor-α (TNF-α) following brain injury [42]. Notably, P2Y6R is over-expressed in inflamed tissues induced by lipopolysaccharide (LPS), the monosodium urate, or TNF-α [43]. P2Y6R signaling facilitates the production of pro-inflammatory chemokines and cytokines, through both autocrine and paracrine circuits in several cell types ex vivo [44]. Warny and colleagues have shown that P2Y6R regulates chemokine production and release in a monocytic cell line [45]. Indeed, UDP activates IL-8 production in THP-1. Furthermore, the authors demonstrate that IL-8 expression is enhanced after LPS administration in THP-1 cells overexpressing P2Y6 [45]. It has been demonstrated that UDP- and LPS-induced IL-8 release from human monocytic THP-1 cells is mediated by an autocrine stimulation of the P2Y6R [45]. Another study showed that blocking P2Y6R could prevent LPS-induced cell death in vivo and in vitro [46].

On the contrary, Wen and colleagues observed that inflammatory cytokine mRNA expression of IL-1α, IL-1β, IL-6, IL-10, TNF-α and transforming growth factor-beta (TGF-β), both in the in vivo model of stroke and in vitro, did not change with MRS2578, which is a selective P2Y6R antagonist [47]. This indicates that P2Y6R inhibition only decreased the phagocytic function of microglia, without affecting the inflammatory response [47].

ATP is a potent inducer of microglia motility [48]. It activates both chemotactic and chemokinetic activities via P2X4R stimulation. Both ATP-gated P2X4R and UDP-activated P2Y6R are up-regulated in activated microglia following neuronal injury [49]. UDP-induced P2Y6R stimulation can prevent the ATP-dependent migration of microglia, most likely by switching from its migratory phenotype to a phagocytic one [50,51,52]. Briefly, Bernier and co-authors reported that activation of P2Y6 by UDP inhibits ATP-evoked P2X4R responses, in both resting and activated microglia [50]. For instance, in resting as well as LPS-activated primary microglia, P2Y6R decreases P2X4R-mediated calcium entry and inhibits the dilation of P2X4R channels into a large-conductance pore [50]. Furthermore, P2Y6R activation modulates the ATP-dependent migration of microglia, a process likely involved in their shift from the migratory to phagocytic phenotype. Moreover, P2Y6R activation decreased P2X4R current amplitude, activation, and desensitization rates, and reduced P2X4R channel permeability to the large cation N-methyl-D-glucamine-1 (NMDG1) [50]. The authors suggested that this inhibitory crosstalk was caused by phospholipase C-mediated hydrolysis of the phosphoinositide PI(4,5)P2, which is a necessary cofactor for the P2X4R channel function [50].

Autophagy is a self-degradative process that is involved in cellular homeostasis and is required to maintain normal cellular physiology under stressful conditions [53,54]. It is well established that autophagy and phagocytosis are both lysosomal degradation pathways, with some similarities [55,56]. Pharmacological and genetic evidence suggests that autophagy functions pleiotropically in cellular homeostasis, growth, survival, and differentiation [57,58]. Monocytes have the remarkable ability to migrate to tissues in reaction to inflammation, where they are subjected to differentiation into morphologically and functionally heterogeneous cells, such as macrophages, dendritic cells, and osteoclasts. The differentiation of human peripheral blood monocytes into macrophages can be replicated ex vivo through exposure to colony-stimulating factor-1 (CSF-1, also known as M-CSF), a mechanism involving selective activation of caspase-8 and caspase-3 [59]. CSF-1 binding to the CSF-1R receptor activates consecutive AKT (also known as protein kinase B, PKB), resulting in the formation of a caspase-8 triggering platform and the differentiation of monocytes into macrophages [60]. Recently, it has been reported that P2Y6R mediated autophagy and monocyte differentiation. Briefly, the physiological P2Y6R ligand UDP and the specific P2Y6R agonist MRS2693 restore normal monocyte differentiation through the re-induction of autophagy in primary myeloid cells from chronic myelomonocytic leukemia (CMML) patients [61].

Beyond its immunologic functions, it has been reported that P2Y6R has a protective effect against TNFα-induced apoptosis [62]. For instance, 1321N1 astrocytoma cells stably transfected with rat P2Y6R are coupled to both phosphatidylcholine-specific phospholipase C (PC-PLC) and phosphatidylinositol-specific phospholipase C (PI-PLC), which protect cells against TNFα-induced apoptosis, through the activation of protein kinase C (PKC) isotypes. Activation of the P2Y6R also stimulates extracellular-signal-regulated kinase (ERK), which is controlled by PKC and is a partial factor in cell protection by UDP against TNFα-induced cell death [62]. Similarly, it has been suggested that the activation of P2Y6R initiates NF-κB signaling and thus improves osteoclast survival [33]. Subsequently, the inhibition of P2Y6R can be effective therapeutically in the treatment of inflammatory bone diseases [33]. NF-κB is a transcription factor that controls multiple physiological functions and is implicated in the pathogenesis of numerous illnesses, and has recently been acknowledged as a target for new anti-inflammatory drugs. However, genetic studies in mice indicate that NF-κB may also be a complicated therapeutic target for inflammatory diseases [63,64,65]. For instance, the NF-κB pathway controls the development of pro-inflammatory cytokines, the recruitment of leukocytes or cell survival, which are essential contributors to the inflammatory response, and maintains the inflammatory response by persistent leukocyte activation [66]. On the other hand, NF-κB may enhance leukocyte apoptosis in certain circumstances, and lead to the resolution of inflammation [66]. In other words, P2Y6R has multiple functions that depend mainly on the context of the disease.

## 5. P2Y6R as a Potential Target in Neurological Diseases

P2Y6Rs have been reported to convey essential functions in CNS disorders [30]. In recent years, growing research in animal models and human tissues has indicated the involvement of P2Y6R in the pathogenesis of multiple CNS disorders. In the next section, we will describe the role of UDP/P2Y6R signaling in ischemic stroke, AD, PD, radiation-induced brain injury, and neuropathic pain. Hence, P2Y6R is a possible therapeutic target by improving debris and pathogen phagocytosis, which facilitates repair.

### 5.1. Ischemic Stroke

Ischemic stroke is one of the leading causes of mortality and permanent illness, due to widespread cell loss and massive pathogenic alterations [67]. Microglial activation is one of the hallmarks of acute stroke. They first respond to the damage and are recruited during an ischemic stroke into the region of the infarction [68]. They act by producing a cascade of pro-inflammatory or anti-inflammatory cytokines [69]. Activated microglia can secrete a variety of pro-inflammatory cytokines to worsen blood-brain barrier (BBB), disruption and tissue injury [70]. On the contrary, they promote tissue repair by phagocytosis and the production of neurotrophic factors [71].

P2Y6R-mediated microglial phagocytosis has been reported to be beneficial for debris clearance and functional recovery after an ischemic stroke [47]. Briefly, the P2Y6R levels increased significantly in ischemic mice after transient middle cerebral artery occlusion (tMCAO), and the P2Y6R seems to be expressed exclusively in microglia [47]. Moreover, inhibition of P2Y6R activity exacerbated neurological function deficit and brain injury after tMCAO. For instance, the P2Y6R activity was blocked by the intraperitoneal injection of the selective P2Y6R antagonist MRS2578 (3 mg/kg) for three consecutive days, after 90 min of tMCAO [47]. TUNEL cells (apoptotic cells) engulfed by Iba1+ microglia are decreased after the P2Y6R antagonist in the peri-infarct region in the MRS2578 group compared to the control group [47], suggesting that the MRS2578 significantly inhibited microglial phagocytosis in the brain after tMCAO [47]. Inhibiting the P2Y6R activity did not affect the expression of inflammatory cytokines and neutrophil infiltration. The results showed that the mRNA levels of IL-1α, IL-1β, IL-6, IL-10, TNF-α, and TGF-β are up-regulated after three days of tMCAO, except IL-10 [47]. However, the expression of these aforementioned cytokines remained similar in the MRS2578 and control groups [47]. Such results suggest that P2Y6R -mediated microglial phagocytosis plays a protective effect during the acute stage of ischemic stroke, which could be a therapeutic target for ischemic stroke. However, further studies are needed to elucidate the mechanism. In addition, the effect of P2Y6R inhibition on the late stage of ischemic stroke has yet to be uncovered.

### 5.2. Alzheimer’s Disease

Alzheimer’s disease (AD) is the primary cause of dementia worldwide; a progressive, incurable neurodegenerative disease that is associated with chronic activation of innate immunity within the CNS [72]. AD is characterized pathologically by the accumulation of extracellular amyloid-β (Aβ) 1–42 peptide and intracellular neurofibrillary tangles that contain hyperphosphorylated tau [73], together with pathological gliosis, inflammation, neuritic dystrophy and neuronal loss [74]. Many hypotheses have been proposed to clarify AD pathogenesis, including different pathological processes, such as amyloid aggregation, tau protein hyperphosphorylation, dysregulation of metal ions, and persistent neuroinflammation [75]. Although it has previously been proposed that neuroinflammatory events play a crucial role in the development of AD [76], this is no longer the case, since these events are involved in the clearance of Aβ. It is a lack of proper inflammatory response by microglia that allows Aβ accumulation in the brain of AD patients and mouse models of AD [77]. Activated microglia display a systematic series of morphological changes, gene expression, and produce and release numerous chemical mediators, including pro-inflammatory cytokines that can produce immunological actions and modify neuronal function [78]. Accumulating data recently identified essential functions for ATP receptors of activated microglia in AD [75].

UDP activates P2Y6R, which is released from stressed or injured neurons [36]. P2Y6R activation increased the clearance of amyloid plaques by CD11b-positive microglial cells in the AD mouse model PS1/APP, suggesting that P2Y6R-mediated microglial phagocytosis contributes to Aβ clearance [7]. Additionally, P2Y6R activation preserved synaptic plasticity and reversed contextual hippocampal-dependent memory in PS1/APP mice [7]. Microglial P2Y6R stimulation contributes to a decrease in Aß burden, which improves synaptic and cognitive deficits that are AD hallmarks. Microglial P2Y6R has been shown to control neuronal debris phagocytosis that should minimize neuroinflammation in the AD brain, although the same pathway may facilitate the phagocytosis of viable neurons that could lead to neurodegeneration [79]. By blocking P2Y6R with the small molecule antagonist MRS2578, neurons were spared from an untimely death following intracerebral injection of LPS into rats. In culture, MRS2578 protected neurons exposed to Aβ from being eaten by overzealous microglia [46]. Moreover, PY26R deficiency spared mice from neuronal loss and memory deficits induced by injection of Aβ oligomers, by tau pathology, or by the aging process itself [7]. Collectively, further experiments are required to determine the effectiveness of multiple P2YR targets for therapy.

### 5.3. Parkinson’s Disease

Parkinson’s disease (PD) is a neurodegenerative disease that affects nearly 3% of elder individuals, characterized by progressive loss of dopaminergic neurons of the substantia nigra pars compacta (SNpc), with a decrease of dopamine concentration in the striatum, and the presence of protein aggregates positive for α-synuclein, known as Lewis bodies [80,81]. McGeer’s research team has proposed that inflammation could be the first pathogenic pathway of PD [82]. Although neuronal loss has been established as proof of the ongoing inflammation of the CNS, multiple research studies have documented microglial activation, cytokine production, and the involvement of autoantibodies that univocally indicate inflammatory processes in PD [83,84]. Vast amounts of ATP released into the extracellular space by dying cells stimulate purinergic receptors, which can play a significant role in PD-related neurodegeneration [29]. It has been reported that P2Y6R mRNA levels in PD patients were significantly higher than in the healthy controls [85]. Yang et al. found that activated microglia could up-regulate P2Y6R expression [85]. In addition, the change in P2Y6R expression was accompanied by the upregulation of inflammatory cytokines [85]. The authors further revealed the role of P2Y6R in the regulation of microglial function, as it could not only initiate microglial phagocytosis, but also actively participate in the process of microglia-induced inflammation [85]. P2Y6R blocking could be a possible therapeutic option for the treatment of PD patients, by inhibiting neuroinflammation triggered by microglia [85]. However, the exact contribution of inflammation in PD has yet to be determined, and it is likely to be a consequence of neuronal loss, and not a direct cause of the disease.

### 5.4. Radiation-Induced Brain Injury

Tumors that affect the head, neck, and brain, as well as brain metastases that arise in 20–40% of cancer patients, account for significant morbidity and mortality [86]. Radiation is one of the most successful therapeutic strategies for such tumors [87]. Radiotherapy, while helpful in treating CNS and tumors of the head and neck, may inflict catastrophic damage to healthy CNS tissues [88]. Although the precise pathogenic pathways of radiation-induced brain injury remain widely unclear, studies have shown that microglia may play a crucial role in releasing pro-inflammatory factors that cause an inflammatory response when stimulated by radiation [89,90]. Besides this inflammatory reaction component, microglia are often skilled phagocytes in the CNS, maintaining the CNS homeostasis [91]. Activated and phagocytic microglia also accumulate at the lesion/spinal cord interface in an irradiated hemi-sectioned spinal cord [92]. The pathways underlying microglial phagocytosis in the pathogenesis of radiation-induced brain injury are still far from being understood. It was also found that extracellular uridine UDP produced by damaged cells triggers microglial phagocytosis [38]. Xu and others have shown that microglial phagocytosis was triggered after radiation, both in vitro and in vivo [39]. Radiation increased the expression of P2Y6R, and a P2Y6R-specific antagonist blocked this increase [39]. Radiation caused neuronal apoptosis and induced demyelination, both of which were exacerbated by inhibiting UDP/P2Y6R signaling and microglial phagocytosis [39]. Moreover, the authors determined that the Ras-related C3 botulinum toxin substrate 1 (Rac1)-myosin light chain kinase (MLCK) pathway was involved in P2Y6R-mediated microglial phagocytosis in radiation-induced brain injury [39]. Collectively, UDP/P2Y6R is implicated in the pathogenesis of radiation-induced brain damage, by triggering the activation of phagocytosis by microglia, which is associated with the release of pro-inflammatory cytokines.

### 5.5. Neuropathic Pain

Neuropathic pain (NP) refers to pain caused by nervous system disorders or injuries, the specific mechanism of which remains unknown [93,94]. Hence, the investigation of pathogenesis and therapeutic approaches for NP is desperately required. It is well known that neuroinflammation plays a significant role in maintaining NP, primarily via mediating cerebral sensitization [95]. Notably, the crosstalk between microglia and neurons is a central component driving neuroinflammation, and inhibiting microglial activity may contribute to pain alleviation [96]. Bian and colleagues developed an NP model using Sprague–Dawley (SD) rats undergoing chronic sciatic nerve constriction injury (CCI), to evaluate the dynamic expression of P2Y6R, IL-6, and JAK2/STAT3 pathway proteins during NP development [97]. Moreover, the authors determined the expression of microglial marker Iba-1 and corresponding morphological changes of microglial cells [97]. The administration of the P2Y6R antagonist resulted in the inhibition of microglial polarization and IL-6 production, contributing to a reduction of NP in CCI rats [97]. Accordingly, this evidence showed a crucial function of P2Y6R in modulating the JAK2/STAT3-mediated nociceptive transmission and microglial activation [97]. Collectively, UDP/P2Y6R mediated microglial activation resulted in neuroinflammation, which exacerbated NP. Therefore, the inhibition of P2Y6R may serve as a potential target for NP therapy in future studies.

## 6. Current Limitations and Prospects

While several antagonists of P2Y6R have been produced and evaluated, the chemical structures of these molecules are limited. MRS2578 is the most effective and selective inhibitor available at present, but it binds irreversibly, has restricted stability in aqueous solution, and has limited in vivo efficacy [98]. P2Y6R is a G-protein coupled receptor. Ironically, there are increasing numbers of GPCRs that have been described as having poorly validated detection reagents and having seriously deficient antibodies [99]. Three commercially available antibodies to P2Y6R have been tested to validate the specificity of these antibodies by performing immunohistochemistry (IHC) and Western blot (WB) on bladder tissues from P2Y6R knockout mice [99]. The Abs showed a lack of specificity. For instance, in WB, all three antibodies bound similar proteins in both wild type and P2Y6R knockout tissues [99]. Likewise, the immunostaining of both wild type and knockout tissue sections also displayed no difference in staining patterns and intensity [99]. Therefore, P2Y6R antibody-based data need to employ one or more of the rigorous controls.

P2Y6R is a multifaceted receptor that participates in many physiological and pathological conditions, by regulating cellular responses in both immune and non-immune cells. P2Y6R may have distinct and conflicting results in CNS diseases, becoming an angel or a devil based on its activation level, the type of cell becoming investigated, and the nature and the course of the disease. Overall, the signaling of UDP-P2Y6R stimulates the immunity in the context of CNS disorders, induces inflammatory mediators in phagocytic cells, and modulates adaptive immune responses. However, the activation of P2Y6R may create both protective and deleterious responses, depending on the nature of the illness. The findings of the experiments—aimed at studying P2Y6R pathways and utilizing P2Y6R agonists, antagonists, and P2Y6R KO mice—should be carefully analyzed and discussed, in order to accurately bring up the biology and relevance of this receptor in the context of neuro-inflammatory and neurodegenerative diseases. Furthermore, the particularities of each CNS disorder should be considered in the development of pharmacological treatments for P2Y6R.

## 7. Conclusions

P2Y6R is implicated in the pathogenesis of numerous CNS disorders, and has recently been acknowledged as a target for phagocytosis mitigation. However, it may also be a complicated therapeutic target for neuroinflammatory and neurodegenerative diseases. On the one hand, the P2Y6R pathway controls the development of pro-inflammatory cytokines, recruitment of leukocytes, or cell survival, which are essential contributors to the inflammatory response, and maintains the inflammatory response by persistent activation of leukocyte [41,100]. On the other hand, P2Y6R may enhance phagocytosis, leading to the resolution of inflammation and improve tissue repair and healing [101]. In summary, UDP/P2Y6R is implemented in promoting microglia phagocytosis to contain microbial threats and removal of apoptotic cells and tissue debris, such as myelin debris in demyelinating diseases. In conjunction, these phagocytic activities elicit inflammation by releasing pro-inflammatory cytokines, orchestrating the infiltration of additional immune cells, which may exacerbate the inflammatory reaction. Therefore, the modulation of microglial phagocytosis without a persistent excessive inflammatory response could be a potential therapeutic approach. Future research to evaluate the status of these diverse roles for P2Y6R in mediating the neuroinflammation-associated with neurodegenerative diseases are required to determine if this pathway could be a therapeutic target, and in which context.

## Abbreviations

Abbreviations	Definition
CNS	Central nervous system
DAMPs	Disease-associated molecular patterns
ATP	Adenosine triphosphate
UTP	Uridine triphosphate
GPCRs	G protein-coupled receptors
IP_3_	Inositol trisphosphate
Il-6	Interleukin-6
LPS	Lipopolysaccharides
Rac-1	Ras-related C3 botulinum toxin substrate 1
AKT	Protein kinase B
PC-PLC	Phosphatidylcholine-specific phospholipase C
PKC	Protein kinase C
tMCAO	Transient middle cerebral artery occlusion
Iba1	Ionized calcium-binding adaptor-1
Aβ	Amyloid-Beta
SNpc	Substantia nigra pars compacta
NP	Neuropathic pain
CCI	Chronic constriction injury
IHC	Immunohistochemistry
PAMPs	Pathogen associated molecular patterns
TLRs	Toll-like receptors
ADP	Adenosine diphosphate
UDP	Uridine diphosphate
PLC	Phospholipase C
IL-1	Interleukin-1
TNF-α	Tumor necrosis factor
TGF-β	Transforming growth factor-beta
M-CSF	Macrophage colony-stimulating factor
CMML	Chronic myelomonocytic leukemia
PI-PLC	Phosphoinositide-specific phospholipase C
ERK	Extracellular signal-regulated kinases
AD	Alzheimer’s disease
TUNEL	Terminal deoxynucleotidyl transferase dUTP nick end labeling
PD	Parkinson’s disease
MLCK	Myosin light chain kinase
SD	Sprague–Dawley
WB	Western blot
NMDG1	N-methyl-D-glucamine-1
